# A Surgicel™ Surprise: Retained Oxidized Regenerated Cellulose Posing as a Biloma With Retained Gallstones After Subtotal Cholecystectomy

**DOI:** 10.7759/cureus.90234

**Published:** 2025-08-16

**Authors:** Rami Madani, David M Richter, Dakota De Cecco, Andrew Alfred, Saad Shebrain

**Affiliations:** 1 Department of Surgical Sciences, Western Michigan University Homer Stryker M.D. School of Medicine, Kalamazoo, USA; 2 Department of Internal Medicine, University of Michigan Medical School, Ann Arbor, USA; 3 Department of Anesthesiology, West Virginia University School of Medicine, Morgantown, USA

**Keywords:** biloma, retained gallstones, subtotal laparoscopic cholecystectomy, surgicel, surgiceloma

## Abstract

Surgicel™ is a bioabsorbable local hemostatic agent frequently used to control bleeding during laparoscopic cholecystectomy. Although rare, residual Surgicel™ can cause a local inflammatory response and subsequent calcification. This may complicate the interpretation of imaging studies, leading to misdiagnosis and unnecessary workup.

Sixteen months after subtotal cholecystectomy for gangrenous cholecystitis, a 64-year-old man presented to the emergency department with epigastric pain. A CT of the abdomen demonstrated mild inflammation and a localized hyperdensity within the residual gallbladder, suggestive of biloma and retained gallstones. However, a subsequent hepatobiliary iminodiacetic acid scan showed normal post-cholecystectomy changes, ruling out biloma. Accordingly, the patient’s clinical presentation and initial radiologic findings were consistent with calcification of retained Surgicel™.

Calcification of Surgicel™ is rare and radiographically mimics gallstones. A thorough evaluation of the patient’s complete operative history is critical to avoid misdiagnosis and unnecessary interventions.

## Introduction

Surgicel™ absorbable hemostat (oxidized regenerated cellulose (ORC)) is a bioabsorbable thrombogenic agent that is frequently used during surgery to control bleeding from small vessels, particularly in scenarios where conventional hemostasis can otherwise be difficult to achieve. Accordingly, Surgicel™ is especially helpful in laparoscopic surgery, where even minor bleeding can be problematic due to the narrow surgical field ​[1]. Although the manufacturer offers various Surgicel™ formulations, Surgicel™ Original appears to be the most used in laparoscopic cholecystectomy ​[2]. In addition to providing a physical matrix for clot formation, this woven ORC mesh binds directly to hemoglobin, promoting platelet aggregation ​[3]. Typically, Surgicel™ is completely reabsorbed by the body within 7-14 days; however, in rare cases, residual Surgicel™ can be retained, causing a localized foreign body reaction, a “surgiceloma.” In such cases, patients can exhibit a range of clinical and radiologic features, which may evolve over time.

In the early postoperative period (days to weeks), patients may present with fever, abdominal pain, and signs of infection. At this stage, the surgiceloma may be seen on CT as a hyperdense mass with a “whorled” appearance. Additionally, there may be air pockets within the mass, and associated inflammatory changes are often observed in the surrounding tissue. This may complicate diagnosis, as the surgical surgiceloma may mimic a postoperative abscess. As time progresses (weeks to months), the surgiceloma begins to appear less dense and more heterogeneous on CT imaging [4]. MRI can be especially useful at this phase, as Surgicel™’s characteristic appearance on T1- and T2-weighted images helps differentiate it from other postoperative complications, such as abscesses or neoplasms ​[5,6].

During the late postoperative period (months to years), retained Surgicel™ can lead to chronic inflammatory reactions, granuloma formation, and subsequent calcification. Due to a wide variation in symptomology and radiographic findings, chronically retained Surgicel™ presents a much more challenging diagnosis ​[7]. This is especially the case in the context of hepatobiliary surgery, as the only long-term study of retained Surgicel™ involved sonography in post-thyroidectomy patients ​[8]. In such cases, diagnosis requires physicians to perform a thorough review of the patient’s prior surgical records to correlate Surgicel™ use with imaging findings. Physicians must be able to identify retained Surgicel™ in both the early and late postoperative periods, as misdiagnosis may lead to unnecessary and invasive interventions. This is especially important, as this appears to be an exceptionally rare phenomenon. An extensive review of the PubMed database revealed no previously published reports of retained Surgicel™ mimicking a biloma following subtotal cholecystectomy. To our knowledge, this case represents the first documented instance of residual Surgicel™ masquerading as a biloma with retained gallstones on imaging in the late post-cholecystectomy period.

## Case presentation

A 64-year-old male with a history of stage 4 chronic kidney disease, hypertension, hyperlipidemia, coronary artery disease, type II diabetes mellitus, and obesity presented to the ED with right upper quadrant and epigastric pain, nausea, and retching.

**Figure 1:**
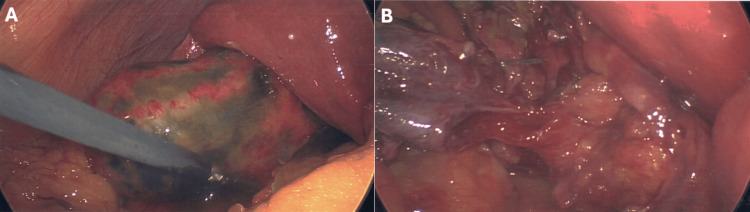
Photographs obtained intraoperatively when the patient’s initially presented with acute gangrenous cholecystitis Due to significant inflammation and patchy gallbladder wall necrosis (A), a fenestrated subtotal cholecystectomy was successfully performed (B).

The patient had a history of two prior abdominal surgeries. The patient underwent a laparotomy following a gunshot wound sustained four decades prior; a bullet was retained in the right lobe of his liver. Sixteen months prior, the patient underwent a fenestrated subtotal cholecystectomy (FSC) for complicated cholelithiasis and acute-on-chronic cholecystitis. Intraoperatively, the surgeons observed severe inflammation, significant adhesions, and gangrenous cholecystitis (Figure 1A). Although a total cholecystectomy was initially planned, it was determined that a total cholecystectomy could not be achieved safely, given the severity of inflammation and adhesions obscuring the natural safety planes. At that time, the surgeons elected to perform an FSC, wherein the anterior wall of the gallbladder is removed, and the remaining gallbladder is left open ​[9]. Cholesterol stones were removed from the remnant gallbladder; the cystic duct was examined and determined to be free of stones (Figure 1B). Notably, the surgeons encountered hemorrhage into the gallbladder fossa and significant venous oozing from the mucosa of the infundibular portion of the remnant gallbladder. Given the close proximity to critical structures, electrical hemostasis was avoided; instead, two large sheets of Surgicel™ Original were placed in the gallbladder fossa and the remnant portion of the gallbladder.

Additionally, a Jackson-Pratt drain was placed in the gallbladder fossa. Postoperatively, the patient was placed on parenteral ceftriaxone and metronidazole for two days and was discharged home on oral ciprofloxacin and metronidazole. The patient recovered uneventfully and was discharged home on postoperative day (POD) 3, and the drain was removed on POD 7.

**Figure 2:**
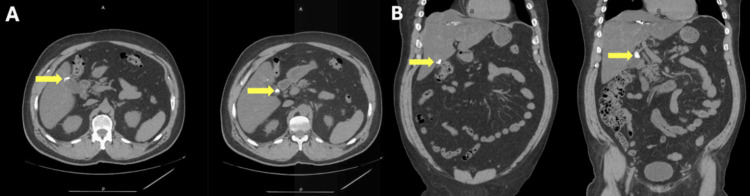
Axial (A) and coronal (B) CT of the abdomen Hyperdense masses in an apparently full gallbladder fossa (yellow arrows) with local inflammatory changes. Of note, the additional hyperdense mass in the right lobe of the patient’s liver was consistent with a retained foreign body resulting from penetrating trauma years prior. CT: computed tomography

**Figure 3:**
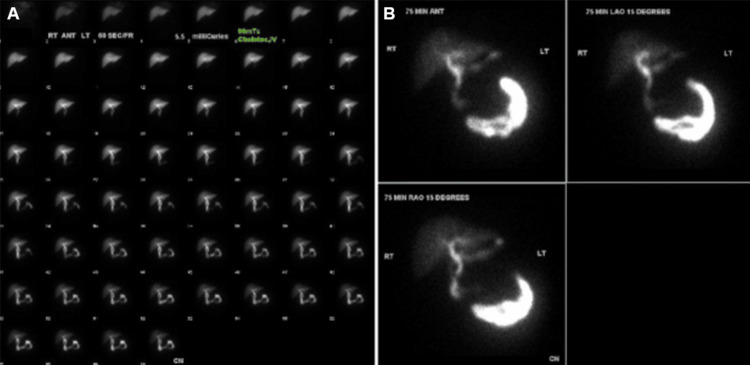
HIDA scan HIDA scan demonstrating normal uptake and excretion of radiotracer through the hepatobiliary system without evidence of tracer extravasation. Dynamic sequential images (A) show normal transit of radiotracer into the small bowel. Delayed static images at 75 minutes (B) in ANT, LAO (15°), and RAO (15°) projections confirm intact post-cholecystectomy clips and exclude biloma, with radiotracer confined to expected biliary and intestinal pathways. ANT: anterior, LAO: left anterior oblique, RAO: right anterior oblique, HIDA: hepatobiliary iminodiacetic acid

When the patient presented 16 months later to the ED, radiologic evaluation included a CT scan of the abdomen, which demonstrated high-density calcifications within the remnant neck of the gallbladder and mild local inflammation (Figure 2). This complex presentation was initially thought to be most consistent with a possible biloma with retained gallstones. Alternative diagnoses considered included abscess, hematoma, and neoplasm. The patient underwent ultrasound imaging, and a hepatobiliary iminodiacetic acid (HIDA) scan was ordered to check for biloma and retained gallstones. Interestingly, the HIDA revealed intact post-cholecystectomy clips without evidence of tracer extravasation, effectively ruling out biloma (Figure 3). A thorough, exhaustive review of the patient’s operative records, in addition to the unremarkable HIDA scan, facilitated a proper diagnosis of retained Surgicel™ (surgiceloma) in the late postoperative period. Following a multidisciplinary care team discussion, including a radiology consultation confirming the presence of calcified Surgicel™, and in light of the patient’s normal and reassuring laboratory results, no further treatment was pursued. On the last visit to the surgery clinic, the patient was doing well and required no further follow-up.

## Discussion

When a critical view of safety cannot be obtained during laparoscopic cholecystectomy, subtotal cholecystectomy is often employed as a damage-control approach to minimize the risk of bile duct injury. Common indications include hepatic cirrhosis, gangrenous cholecystitis, severe chronic cholecystitis with dense adhesions, aberrant anatomy within Calot’s triangle, and certain presentations of Mirizzi’s syndrome. Although subtotal cholecystectomy has a comparable overall complication rate to total cholecystectomy, it is associated with a higher incidence of specific postoperative complications, including retained gallstones, bile leaks, and localized fluid collections. Bile leaks, while often managed effectively with endoscopic retrograde cholangiopancreatography and biliary stenting, can be serious if undetected. While it is relatively uncommon for bile leaks to occur several months after subtotal cholecystectomy, the development of localized fluid collections, such as bilomas or seromas, in the residual gallbladder fossa is not unexpected. In rare instances, as seen in our patient, retained Surgicel™ can mimic retained gallstones or abscess within the gallbladder fossa. Recognition of this possibility underscores the importance of correlating imaging findings with a detailed surgical history to avoid misdiagnosis and unnecessary further intervention.

To our knowledge, this case represents the first documented instance of calcified residual Surgicel™ masquerading as biloma and retained gallstones during the late postoperative period. This signifies a novel radiologic appearance of surgiceloma in patients who have undergone subtotal cholecystectomy. Our case showed a hyperdense, non-enhancing mass within the gallbladder fossa, notably lacking the focal gas patterns commonly associated with acutely retained Surgicel™ ​[10,11]. This likely reflects the patient’s late presentation, as, over time, soft tissue reaction can result in the formation of a well-demarcated granuloma with dystrophic calcifications. On a microscopic level, the Surgicel™ lattice appears to act as a scaffold for tissue ingrowth over time, occasionally leading to calcification or mass formation ​[12].

The long-term persistence of Surgicel™ in our patient may be attributed to several contributing factors. Surgicel™ has an absorption time of two to seven days and complete degradation by eight weeks ​[13]; however, placement of large quantities in relatively avascular spaces, such as the gallbladder fossa or remnant gallbladder, can significantly delay resorption ​[14]. Current literature recommends removing excess Surgicel™ once hemostasis is achieved to minimize this risk ​[15]. Additionally, our patients’ comorbidities, including diabetes mellitus, chronic kidney disease, and obesity, may impair local wound healing and promote chronic inflammatory responses, increasing the risk of foreign body granuloma formation. While individual susceptibility to foreign body reaction may vary, current evidence does not clearly define genetic or immunologic predictors of surgiceloma formation. These considerations underscore the need for judicious use of hemostatic agents, particularly in patients with impaired healing capacity.

## Conclusions

This case emphasizes the necessity for a broad differential diagnosis when evaluating postoperative patients despite the routine nature of surgical management for gallstones. Accurate interpretation of imaging findings requires careful consideration of the patient’s surgical and medical history, particularly in those with prior subtotal cholecystectomy. Unnecessary surgical intervention remains a significant risk when postoperative changes are misinterpreted as pathology. This underscores the importance of multidisciplinary awareness among surgeons, radiologists, and general physicians that hemostatic agents applied intraoperatively, such as Surgicel™, may persist and mimic abscesses, neoplasms, hematomas, or calcified intra-abdominal masses, even in the chronic postoperative period. Recognizing this possibility is critical to minimizing misdiagnosis and avoiding avoidable reoperation in patients with complex surgical histories.
